# Correcting for the influence of sampling conditions on biomarkers of exposure to phenols and phthalates: a 2-step standardization method based on regression residuals

**DOI:** 10.1186/1476-069X-11-29

**Published:** 2012-04-26

**Authors:** Marion Mortamais, Cécile Chevrier, Claire Philippat, Claire Petit, Antonia M Calafat, Xiaoyun Ye, Manori J Silva, Christian Brambilla, Marinus JC Eijkemans, Marie-Aline Charles, Sylvaine Cordier, Rémy Slama

**Affiliations:** 1 Team of Environmental Epidemiology Applied to Reproduction and Respiratory Health, Inserm, Institut Albert Bonniot (U823), Grenoble, France; 2 Inserm, U1061, Montpellier, France; 3 Inserm, U625, Rennes, France; 4 Grenoble University, Institut Albert Bonniot (U823), Grenoble, France; 5 Centers for Disease Control and Prevention, Atlanta, GA, USA; 6 Inserm and Grenoble University, Institut Albert Bonniot (U823), Molecular Basis of Lung Cancer Progression, Grenoble, France; 7 Julius Center for Health Sciences and Primary Care, UMC, Utrecht, The Netherlands; 8 Inserm and INED Joint Research Group, PARIS, and Inserm, U1018, CESP, Villejuif, France

**Keywords:** Biomarker, Endocrine Disruptor, Phenols, Phthalate esters, Pregnancy, Sampling conditions

## Abstract

**Background:**

Environmental epidemiology and biomonitoring studies typically rely on biological samples to assay the concentration of non-persistent exposure biomarkers. Between-participant variations in sampling conditions of these biological samples constitute a potential source of exposure misclassification. Few studies attempted to correct biomarker levels for this error. We aimed to assess the influence of sampling conditions on concentrations of urinary biomarkers of select phenols and phthalates, two widely-produced families of chemicals, and to standardize biomarker concentrations on sampling conditions.

**Methods:**

Urine samples were collected between 2002 and 2006 among 287 pregnant women from Eden and Pélagie cohorts, from which phthalates and phenols metabolites levels were assayed. We applied a 2-step standardization method based on regression residuals. First, the influence of sampling conditions (including sampling hour, duration of storage before freezing) and of creatinine levels on biomarker concentrations were characterized using adjusted linear regression models. In the second step, the model estimates were used to remove the variability in biomarker concentrations due to sampling conditions and to standardize concentrations as if all samples had been collected under the same conditions (e.g., same hour of urine collection).

**Results:**

Sampling hour was associated with concentrations of several exposure biomarkers. After standardization for sampling conditions, median concentrations differed by ‒ 38 % for 2,5-dichlorophenol to +80 % for a metabolite of diisodecyl phthalate. However, at the individual level, standardized biomarker levels were strongly correlated (correlation coefficients above 0.80) with unstandardized measures.

**Conclusions:**

Sampling conditions, such as sampling hour, should be systematically collected in biomarker-based studies, in particular when the biomarker half-life is short. The 2-step standardization method based on regression residuals that we proposed in order to limit the impact of heterogeneity in sampling conditions could be further tested in studies describing levels of biomarkers or their influence on health.

## Background

Diesters of phthalic acid (phthalates) and some phenols are man-made chemicals widely used in personal care and consumer products. Some of these compounds are endocrine-disruptors and can impact various health outcomes in animals [1,2], which raises concern about their potential health impacts in humans. Widespread exposure has been documented in pregnant women [3-5], who deserve specific consideration because of concerns on the effects of exposure to endocrine disruptors during intra-uterine life [6,7].

A common approach for investigating human exposure to these compounds is the measurement of urinary concentrations of phthalates metabolites [8] and of the sum of concentrations of free and conjugated forms of phenols [9].

Phthalates and phenols are non persistent compounds in humans with a half life in non-pregnant subjects generally estimated to be below 24 hours [10,11]. A high day-to-day variability in their urinary concentrations has been documented among non pregnant [12] and pregnant [13] women, as well as within-day variations for some phthalates metabolites [14,15] and for bisphenol A (BPA) [16], but not for triclosan [17]. Other potential sources of variability in biomarker levels that can be seen as nuisances include duration of storage of the biological sample at room temperature and between-subject variations in gestational age at urine sampling or in time elapsed since last urine void.

Making sampling conditions identical across study participants is a way to limit this nuisance and make biomarker levels a better proxy of short-term personal exposure to these compounds in descriptive or etiologic studies [18]. However, for large-scale observational studies, some degree of variation with the sampling protocol (e.g., in hour of urine collection) is hardly avoidable for some participants. Unless one is interested in the average exposure of the population as a whole (in which case maximizing variability in hour of sampling might be a good option for quickly metabolized compounds), this variability is a potential source of nuisance. Because excluding participants not strictly adhering to the sampling protocol might induce selection bias, alternative approaches allowing to statistically standardize measured concentrations are worth investigating.

Such approaches have to our knowledge rarely been applied. In purely descriptive studies, assayed biomarker levels are often left untransformed. When studying the impact of biomarker levels on health outcome, adjusting for sampling conditions influencing biomarker levels is sometimes performed. This approach may not be efficient because adjusting for sampling conditions in a regression model aiming at characterizing the effect of exposure on disease risk is unlikely to correct for the effect of sampling conditions on biomarker levels. As an illustration, in a study aiming at characterizing the association between serum concentration of 25-hydroxyvitamin D and cancer risk where between-subject differences in season of collection of blood sample existed, Wang et al. [19] considered several ways to handle differences in this sampling condition influencing the biomarker level. They have shown that, because of seasonal variations in 25-hydroxyvitamin D, choosing season-specific cut-offs to categorize the levels of this biomarker was a more efficient approach than adjusting for the date of sampling in a regression model where cancer occurrence was the dependent variable. Choosing season-specific cut-offs for categories of biomarker levels fluctuating with season is, in terms of identifying the group with the highest estimated exposure, equivalent to correcting biomarker levels by a value depending on the season of sampling. This approach has the advantage of being applicable independently of any information on health outcome, e.g., in descriptive (biomonitoring) studies.

Here, we generalize this approach to the situation where several sampling conditions are considered simultaneously, using a 2-step standardization method based on regression residuals. The specific objectives of our study were to determine the influence of sampling conditions on select phthalates metabolites and phenols urinary concentrations among pregnant women; we then described the concentrations of phthalates metabolites and phenols standardized for sampling conditions.

## Methods

### Study population

**Figure 1:**
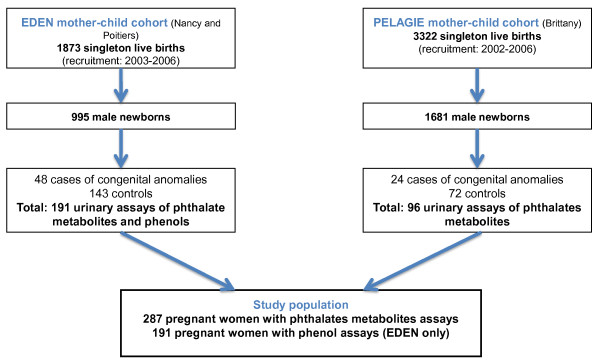
Flow Chart of Study Population, Composed of Pregnant Women from Eden and Pélagie Cohorts, France, 2002 – 2006.

We conducted a case – control study nested within Eden [20,21] and Pélagie [22,23] mother-child cohorts (Figure 1). These cohorts aim to study the effects of fetal and early life events and exposures on health at birth and later in life. Women from the Pélagie cohort (n = 3,421) were enrolled before 19 weeks of gestation (counted from the first day of the menstrual period) from April 2002 to February 2006 in 3 districts of Brittany (France). Women from the Eden cohort (n = 2,002) were enrolled before the end of the 28^th^ week of gestation, from April 2003 to March 2006, at the obstetrical departments of the University Hospitals of Nancy and Poitiers, France. Pregnant women were followed up until delivery, and children are being followed-up. Participants provided informed consent for data and biological sample collection for themselves and their offspring. These cohorts received the approvals of the appropriate ethical committees (Comité Consultatif pour la Protection des Personnes dans la Recherche Biomédicale, le Kremlin-Bicêtre University Hospital, and Commission Nationale de l’Informatique et des Libertés). During pregnancy, women completed questionnaires on socio-demographic characteristics, occupation, and lifestyle. We performed a nested case – control study, including all women (n =72) who delivered boys with external genitalia malformation identified at birth by pediatricians (cases). Three women (controls) were matched to each mother of a case for sex of the baby (i.e., male), center, date of recruitment and gestational duration at the time of collection of the urinary sample, corresponding to 216 controls (Figure 1) [24]. The case – control study aimed at characterizing the impact of phthalates and phenols on congenital malformations [25], but this report focuses on issues related to exposure assessment.

### Urine collection and analysis

For Pélagie cohort, women collected first morning urine void at home between 6 and 19 gestational weeks, as early as possible after recruitment, and mailed it by normal post to the research laboratory, where samples were stored and frozen at − 20 ° C (median storage duration at room temperature, 2 days, Table 1). Mailed vials contained nitric acid in order to avoid bacterial proliferation. For Eden cohort, women were asked to collect first morning urine void at home just before the prenatal study visit, between 24 and 30 gestational weeks, using a polypropylene container (FP40VPS, manufactured by CEB, Angers, France). Women who had forgotten to bring a urine sample collected it in the hospital during the prenatal visit. Samples were aliquoted and frozen at − 80 ° C (median storage duration at room temperature 4 h, Table 1). Time of urine sampling was recorded only for women of Eden.

**Table 1:** Characteristics of French Pregnant Women at the Time of Urine Sampling, and of their Offspring (Eden and Pélagie Cohorts, 2002 – 2006)

	**Eden-Poitiers (n = 91)**		**Eden-Nancy (n = 100)**		**Pélagie-Brittany (n = 96)**			**Overall (n = 287)**
**Characteristics of participants**	**N (%)**	**5^th^-50^th^-95^th^ centiles**	**N (%)**	**5th -50th-95th centiles**	**N (%)**	**5th -50th-95th centiles**	**N (%)**	**5th -50th-95th centiles**
Duration of gestation^a^		37-40-42		37-39-42		36-39-41		36-40-42
Age (years)		21.9-29.0-38.4		20.9-28.9-38.7		23.5-29.6-37.0		22.2-29.3-37.8
BMI ^b^ (kg/m² )		18.4-22.6-33.6		17.1-21.5-31.3		16.8-21.2-24.2		17.7-21.7-32.2
Previous livebirth								
0	38 (42%)		34 (34%)		43 (45%)		115 (40%)	
≥ 1	31 (34%)		44 (44%)		39 (41%)		114 (40%)	
≥ 2	22 (24%)		22 (22%)		14 (14%)		58 (20%)	
Active smoking ( > 0 cig./day)^c^								
No	74 (82%)		84 (84%)		80 (85%)		238 (83%)	
Yes	17 (18%)		16 (16%)		14 (15%)		47 (17%)	
Alcohol ( > 0 glasses/day)^c^								
No	57 (70%)		71 (82%)		79 (82%)		207(78%)	
Yes	24 (30%)		16 (18%)		17 (18%)		57 (22%)	
Maternal education								
High school or less	51 (58%)		44 (45%)		38 (40%)		133 (48%)	
High school + 2 years or more	37 (42%)		53 (55%)		58 (60%)		148 (52%)	
Occupation ^b^								
No	31 (34%)		27 (27%)		12 (12%)		70 (25%)	
Yes	59 (66%)		72 (73%)		84 (88%)		215 (75%)	
Date of urine sampling		Jul. 03- Nov. 04^d^- Aug. 05		Dec.03- Sept.04^d^- Jan.06		Aug. 02- Nov. 03^d^- Mar. 05		Mar. 03- Jun. 04^d^- Nov. 05
Urine Sampling conditions								
Time of sampling								
Before 7.00 AM	12 (18%)		23 (23%)		-		35 (21%)	
7.00 to 7.59 AM	29 (44%)		44 (44%)		-		73 (44%)	
8.00 to 9.59 AM	13 (20%)		27 (27%)		-		40 (24%)	
After 10.00 AM	12 (18%)		6 (6%)		-		18 (11%)	
Creatinine (g/l)		0.46-1.04-2		0.38-1.16-2.30		0.47-1.10-2.1		0.43-1.10-2.20
Gestational weeks at sampling		25-27-28.6		24.1-26.4-28.5		7.4-13-16.9		9.1-25.6-28.3
Duration of storage at room temperature (h)		0.7-3-6		3-5-8		24-48-168		1-5-120
Season of sampling								
Jan-March	27 (30%)		32 (32%)		24 (25%)		83 (29%)	
April-June	22 (24%)		27 (27%)		31 (32%)		80 (28%)	
July-Sept	21 (23%)		17 (17%)		21 (22%)		59 (20%)	
Oct-Dec	21 (23%)		24 (24%)		20 (21%)		65 (23%)	
Day of sampling								
Monday to Friday	91 (100%)		100(100%)		82 (85%)		273(95%)	
Saturday or Sunday	0 (0%)		0 (0%)		14 (15%)		14 (5%)	

^a^ Gestational duration (weeks) at delivery.

^b^ Before pregnancy.

^c^ During pregnancy.

^d^ Median value. November 2004 means that half of the urine samples were collected after November 2004.

In 2008, frozen urine samples were shipped on dry ice to the National Center for Environmental Health laboratory at the Centers for Disease Control and Prevention (CDC) in Atlanta, Georgia (USA). The involvement of the CDC laboratory was determined not to constitute engagement in human subject research. Measurements of 11 phthalate metabolites concentrations (see Additional file 1: Table S1) were conducted using isotope dilution on-line solid-phase extraction-high performance liquid chromatography-electrospray ionization-isotope dilution tandem mass spectrometry [26]. Molar concentrations of 4 metabolites of di(2-ethylhexyl) phthalate (DEHP, see Additional file 1: Table S1) were summed as total DEHP (mol/l). Also, we applied correction factors of 0.66 and 0.72 to the monoethyl phthalate (MEP) and monobenzyl phthalate (MBzP) concentrations, respectively, because the analytic standards used were of inadequate purity [27].

Urinary concentrations of 9 phenols were estimated for the population of Eden only (n = 191) by using a modification of a method involving isotope dilution on-line SPE coupled to high-performance liquid chromatography-tandem mass spectrometry [9]. Phenols were not measured for the Pélagie samples because acidification with nitric acid affected the performance of the analytical method. Assessment of concentrations was not possible for 1 urine sample of a control woman (urine container broken). Total parabens (PB) concentration was calculated by summing butyl-, ethyl-, methyl- and propyl-paraben molar concentrations.

### Statistical analysis

#### Imputation of missing data

Concentrations below the limit of detection (LOD) were replaced by LOD/2^1/2 ^[28]. Missing values in sampling time of day for Eden cohort (n = 25) were imputed using linear regression adjusted for date and sampling season, parity, education level, occupation, active smoking and center. When it was used as an adjustment factor, sampling time was assumed to be 7:00 A.M. for women of Pélagie; models describing the influence of sampling time on biomarker concentrations were estimated excluding Pélagie subjects.

#### Correction for case – control sampling

To make the distribution of biomarker concentrations relevant for the source population (i.e., mothers of male newborns from our cohorts), we corrected for the over-representation of cases induced by our case – control design using a reweighing approach [29]. Center-specific weights corresponded to the inverse of the inclusion probability of controls, so as to give cases and controls the same relative weight than in the original cohorts (about 1 case for 37 male newborns). Unless otherwise specified, this correction was applied in all regression models.

#### Standardization for sampling conditions

We used a 2-step standardization method based on regression residuals to standardize biomarker levels on sampling conditions, that is, to limit the impact of between-subject variations in sampling conditions. The principle is to take away from the observed biomarker concentration a value depending on how much the sampling conditions for subject i differ from the *standard* sampling conditions, i.e. those that should have been observed for all subjects in ideal conditions. This 2-step standardization method is detailed in the Additional file 2 statistical appendix and outlined below:

***First step of standardization: Influence of sampling conditions on biomarker concentrations:*** The first step consists in a description of the association between sampling conditions and the level of each biomarker, adjusted for potential confounders. Sampling conditions considered were hour, season and day of sampling, gestational age at collection, duration of storage of urine sample at room temperature before freezing (in multiples of 24 hours for Pélagie cohort where sampling hour was unknown). We also considered urinary creatinine concentration, seen as a marker of urinary dilution. Creatinine also depends on individual or behavioral characteristics such as muscle mass, and authors have proposed to use specific gravity as a more relevant marker of urinary dilution [13,30]; however this parameter was not available in our study. The association of sampling conditions with the log-transformed concentration ln([Conc]) = Y of each compound was studied using linear regression models adjusted for all sampling conditions simultaneously (*measurement models*). Since individual characteristics were possibly associated to sampling conditions and biomarker concentrations, measurement models were further adjusted for maternal age, body mass index before pregnancy, parity, education, current occupation, active smoking, year of sampling and center.

***Second step of standardization for sampling conditions:*** Using the estimated parameters of the *measurement models*, we predicted the concentrations that would have been observed assuming that all samples had been collected under the same standard conditions. These conditions were assumed to correspond to the median values for hour of sampling (7:30 A.M.), urinary creatinine concentration (1.2 g/l), and time elapsed between sample collection and freezing (5 hours); the day of sampling was assumed to be Monday, the trimester of sampling April-June and the gestational age at collection was assigned as the category corresponding to between 6 and 10 gestational week in Pélagie cohort and to between 24 and 25 week in Eden cohort*.* For each biomarker, this standardized concentration ([Conc^i^]_standardized_) was estimated from the measured concentration [Conc^i^]_measured_ in each subject i using formula (1):

(1) $$\text{ln}({\left[{\text{Conc}}^{i}\right]}^{\text{standardized}}))=\text{ln}({\left[{\text{Conc}}^{i}\right]}^{\text{measured}})–{\text{ ∑ }}_{j}[\beta_{\text{samp cond j}}\times (X_{j}{}^{i}-X_{j}{}^{\text{std}})]\left(1\right)$$

where β_samp cond j_ is the regression parameter quantifying the effect of sampling condition j on the biomarker’s concentration, as estimated in the above-defined measurement model, X_j_^i^ corresponds to the value of this condition for subject i, and X_j_^std^ corresponds to the chosen standard value for sampling condition j. This formula is justified in the Additional file 2 statistical appendix.

Finally, relative variations between median measured and standardized biomarker concentrations were calculated. All calculations were conducted using Stata/SE 10.0.

## Results

The study included 287 pregnant women (Table 1, Figure 1). Eight of the 11 phthalates metabolites and 5 of the 9 phenols were detected in at least 95 % of the population (see Additional file 1: Table S1). Pearson coefficients of correlation between log-transformed biomarker levels were below 0.70, but for the correlations between 2,4-Dichlorophenol (2,4-DCP) and 2,5-DCP (Pearson correlation coefficient of 0.87) and between mono-n-butyl phthalate (MBP) and mono-3-carboxypropyl phthalate (MCPP, correlation 0.76; see Additional file 1: Table S2).

### Sampling conditions and phthalates biomarkers

MBP and MCPP had significantly lower concentrations after 2005 than in 2003 – 2004, while for mono carboxyoctyl phthalate (MCOP) concentrations increased with sampling year. Concentrations of phthalates metabolites tended to decrease with maternal age, in particular for mono-isobutyl phthalate (MiBP). A higher educational level was associated with lower concentrations of MiBP, MBzP and MCOP (see Additional file 1: Table S3).

For Eden cohort, 95 % of urine samples were collected before 10:30 AM. Apart from MEP, phthalates metabolites concentrations tended to decrease with increasing sampling hour (adjusted *P  ≤  0.05* for metabolites of DEHP, MBP, MCPP, mono carboxynonyl phthalate (MCNP) and MCOP, Table 2). Concentrations of all phthalates metabolites increased with urinary creatinine level. Gestational age at sampling was not associated with the urinary concentrations of phthalates metabolites. Only the concentration of MBP decreased with the time elapsed between sample collection and freezing. No phthalate metabolite was associated with either day or season of sampling (Table 2).

**Table 2:** **Adjusted Association Between Log-Transformed Phthalate Monoester Metabolites Urinary Concentrations and Sampling Conditions among French Pregnant Women From Eden and Pélagie Cohorts, 2002 – 2006 **^**a**^

		**DEHP metabolitesh^h^**			**MiBP**		**MBP**		**MCPP**		**MBzP**		**MEP**		**MCNP**		**MCOP**
**Sampling conditions**	**n**	**β**	***P, p trend***	** β**	***P, p trend***	** β**	***P, p trend***	** β**	***P, p trend***	** β**	***P, p trend***	** β**	***P, p trend***	** β**	***P, p trend***	** β**	***P, p trend***
Hour of sampling	191		.02^f^, > .01^g^		.29 ^f^, .08^g^		> 0.01^f ,g^		.04 ^f^, .03^g^		.06 ^f^, .13^g^		.66 ^f^, .71^g^		.12 ^f^, .22^g^		.32 ^f^, .16^g^
> 7.00 AM	36	0		0		0		0		0		0		0		0	
7.00 to 7.59 AM	75	0.00	0.99	− 0.18	0.44	− 0.29	0.22	0.04	0.86	0.33	0.28	− 0.29	0.24	0.26	0.30	0.08	0.73
8.00 to 9.59 AM	59	− 0.39	0.09	− 0.27	0.31	− 0.51	0.05	− 0.08	0.71	− 0.09	0.78	− 0.21	0.46	− 0.16	0.58	− 0.20	0.39
After 10.00 AM	21	− 0.80	0.03	− 0.63	0.06	− 1.08	> 0.01	− 0.88	> 0.01	− 0.68	0.09	− 0.11	0.77	− 0.53	0.29	− 0.39	0.23
Continuous ^b^		− 0.09	0.04	− 0.06	0.22	− 0.20	> 0.01	− 0.10	0.02	− 0.09	0.09	− 0.03	0.62	− 0.12	0.05	− 0.09	0.05
Creatinine(g/l)^c^	287	1.1	> 0.01	0.98	> 0.01	0.96	> 0.01	0.95	> 0.01	1.2	> 0.01	0.96	> 0.01	0.76	> 0.01	0.91	> 0.01
Gestational age (weeks)																	
Pélagie cohort	96		.92 ^f^, .56^g^		.61 ^f^, .34^g^		.74 ^f^, .52^g^		.02 ^f^, .52^g^		.28 ^f^, .24^g^		.23 ^f^, .47^g^		.29 ^f^, .11^g^		.48 ^f^, .36^g^
6-10	24	0		0		0		0		0		0		0		0	
10-12	20	0.03	0.94	− 0.009	0.98	0.16	0.60	0.82	0.01	− 0.27	0.45	− 0.26	0.56	− 0.24	0.47	0.31	0.42
12-14	41	0.06	0.84	0.37	0.29	0.33	0.28	0.21	0.48	0.26	0.44	0.35	0.27	− 0.29	0.38	− 0.10	0.78
14-19	11	0.29	0.56	0.10	0.75	0.06	0.87	− 0.04	0.91	0.22	0.63	− 0.20	0.60	− 0.76	0.07	− 0.20	0.62
Continuous ^d^		− 0.01	0.76	0.02	0.67	0.01	0.74	− 0.008	0.84	0.05	0.36	0.03	0.54	− 0.07	0.17	− 0.04	0.42
Eden cohort	191		.50 ^f^, .30^g^		.25 ^f^, .58^g^		.14 ^f^, .09^g^		.18 ^f^, .08^g^		.01 ^f^, .43^g^		.67 ^f^, .95^g^		.15 ^f^, .36^g^		.63 ^f^, .38^g^
24-25	25	0		0		0		0		0		0		0		0	
25-27	87	− 0.06	0.75	− 0.27	0.23	0.01	0.97	0.05	0.82	− 0.58	0.03	− 0.22	0.44	− 0.20	0.57	− 0.02	0.94
27-30	79	0.13	0.48	− 0.01	0.96	0.38	0.21	0.33	0.14	− 0.06	0.84	− 0.10	0.74	0.15	0.68	0.14	0.49
Continuous ^d^		0.03	0.55	0.04	0.50	0.14	0.04	0.11	0.07	0.07	0.35	− 0.007	0.93	0.07	0.35	0.02	0.79
Day of sampling	287		0.31 ^f^		0.40 ^f^		0.96 ^f^		0.61 ^f^		0.18 ^f^		0.11 ^f^		0.05 ^f^		0.44 ^f^
Monday	57	0		0		0		0		0		0		0		0	
Tuesday	69	− 0.37	0.14	− 0.17	0.42	0.04	0.86	− 0.11	0.54	0.02	0.93	− 0.02	0.92	− 0.29	0.19	− 0.20	0.33
Wednesday	65	− 0.10	0.64	− 0.27	0.11	− 0.0005	0.99	− 0.24	0.20	− 0.06	0.81	− 0.04	0.88	− 0.45	0.02	− 0.39	0.03
Thursday	48	− 0.33	0.13	− 0.18	0.39	− 0.14	0.49	− 0.36	0.11	− 0.11	0.60	− 0.22	0.36	− 0.10	0.73	− 0.30	0.17
Friday	34	− 0.28	0.25	− 0.40	0.04	0.14	0.61	− 0.10	0.73	− 0.53	0.04	− 0.25	0.38	− 0.45	0.02	− 0.47	0.08
Saturday	7	− 0.41	0.17	− 0.04	0.88	0.05	0.85	− 0.42	0.19	0.06	0.85	− 0.92	> 0.01	− 0.63	0.03	− 0.57	0.16
Sunday	7	0.17	0.46	0.17	0.64	− 0.19	0.57	− 0.40	0.21	0.42	0.22	− 0.47	0.20	− 0.18	0.48	− 0.38	0.22
Season of sampling	287		0.54 ^f^		0.34 ^f^		0.92 ^f^		0.86 ^f^		0.52 ^f^		0.84 ^f^		0.81 ^f^		0.98 ^f^
Jan-March	83	0		0		0		0		0		0		0		0	
April-June	80	− 0.05	0.79	− 0.12	0.49	0.08	0.65	− 0.10	0.55	− 0.06	0.75	− 0.12	0.56	0.005	0.98	0.07	0.70
July-Sept	59	− 0.15	0.47	− 0.12	0.57	− 0.004	0.98	− 0.07	0.71	0.21	0.35	0.06	0.82	0.10	0.63	0.06	0.79
Oct-Dec	65	− 0.33	0.15	− 0.38	0.07	0.12	0.62	− 0.17	0.42	− 0.17	0.52	− 0.07	0.76	− 0.10	0.65	0.03	0.90
Duration of urine storage at room temperature (hours)	287		.29 ^f^, .30^g^		.38 ^f^, .29^g^		.82 ^f^, .66^g^		.83 ^f^, .58^g^		.30 ^f^, .27^g^		.51 ^f^, .95^g^		.85 ^f^, .97^g^		.24 ^f^, .38^g^
≤ 3	57	0		0		0		0		0		0		0		0	
3-5	84	− 0.22	0.33	0.29	0.18	0.23	0.34	0.09	0.63	− 0.006	0.99	0.18	0.49	− 0.22	0.43	− 0.12	0.58
6-24	94	− 0.01	0.96	0.13	0.58	0.16	0.57	0.21	0.36	− 0.09	0.80	− 0.21	0.57	− 0.20	0.57	− 0.23	0.39
> 24	52	− 0.35	0.34	0.35	0.26	0.19	0.60	0.20	0.51	0.39	0.38	0.0005	0.99	− 0.05	0.91	0.21	0.55
Continuous ^e^		− 0.002	0.11	− 0.002	0.21	− 0.002	0.05	0.0003	0.86	0.0007	0.69	0.0004	0.81	− 0.001	0.36	0.001	0.43

We applied correction factors of 0.66 and 0.72 to the monoethyl phthalate (MEP) and monobenzyl phthalate (MBzP) concentrations, respectively, because the analytic standards used were of inadequate purity.

^a^ Linear regression models were corrected for over-representation of cases and adjusted for maternal age, BMI, parity, centre, education, occupation and urine sampling conditions.

^b^ Regression parameters are given for an increase by 1 in the sampling hour.

^c^ Regression parameters are given for an increase by 1 g/l in urinary creatinine concentration.

^d^ Regression parameters are given for an increase by 1 week.

^e^ Regression parameters are given for an increase by 1 hour.

^f^*P* of heterogeneity test.

^g^*P* of trend test based on the indicated categories.

^h^ DEHP metabolites: MEHHP + MEOHP + MECPP + MEHP.

### Sampling conditions and phenols biomarkers

A higher educational level was associated with higher parabens concentration (see Additional file 1: Table S4). Sampling hour was negatively associated with BPA concentration (Table 3). Urinary creatinine was positively associated with the concentrations of all phenols but triclosan. BPA concentration increased with gestational age at sampling. It tended to increase with the duration of sample storage at room temperature (Table 3).

**Table 3:** **Adjusted Association Between Log-Transformed Phenol Urinary Concentrations and Sampling Conditions among French Pregnant Women From Eden Cohort, 2002 – 2006 **^**a**^

		**2,4-DCP**		**2,5-DCP**		**Sum of Parabens**		**BP3**		**BPA**		**TCS**	
**Sampling conditions**	**n**	**β**	***P***	**β**	***P***	**β**	***P***	**β**	***P***	**β**	***P***	**β**	***P***
Hour of sampling	191		0.40 ^f^		0.44 ^f^		0.04 ^f^		0.13 ^f^		0.14 ^f^		0.36 ^f^
> 7.00 AM	36	0		0		0		0		0		0	
≥ 7.00 to 7.59 AM	75	0.17	0.51	0.07	0.86	0.02	0.96	− 0.15	0.74	− 0.39	0.05	0.46	0.35
≥ 8.00 to 9.59 AM	59	− 0.14	0.60	− 0.10	0.80	− 0.14	0.79	0.10	0.82	− 0.41	0.04	− 0.29	0.61
After 10.00 AM	21	0.47	0.33	0.82	0.20	1.09	0.12	1.4	0.05	− 0.58	0.04	0.49	0.57
*P* trend			0.73		0.46		0.42		0.11		0.03		0.95
Continuous ^b^		− 0.03	0.65	0.04	0.72	0.07	0.38	0.14	0.31	− 0.07	0.12	− 0.06	0.62
Creatinine (g/l)^c^	191	0.33	0.05	0.36	0.14	0.59	0.04	0.82	> 0.01	0.86	> 0.01	0.20	0.53
Gestational age (weeks)	191		0.90 ^f^		0.96 ^f^		0.52 ^f^		0.13 ^f^		> 0.01 ^f^		0.45 ^f^
24-25	25	0		0		0		0		0		0	
25-27	87	− 0.04	0.90	− 0.11	0.79	− 0.41	0.28	− 0.32	0.40	− 0.06	0.75	− 0.10	0.87
27-30	79	− 0.12	0.70	− 0.12	0.78	− 0.39	0.33	0.35	0.44	0.37	0.07	− 0.51	0.38
*P* trend			0.65		0.81		0.48		0.19		> 0.01		0.26
Continuous ^d^		− 0.08	0.29	− 0.08	0.49	− 0.09	0.41	0.15	0.27	0.14	0.01	− 0.17	0.24
Day of sampling	191		0.51 ^f^		0.49 ^f^		0.86 ^f^		0.79 ^f^		0.91 ^f^		0.76 ^f^
Monday	28	0		0		0		0		0		0	
Tuesday	54	0.52	0.15	0.82	0.10	0.32	0.48	0.17	0.74	− 0.15	0.42	0.41	0.47
Wednesday	54	0.23	0.46	0.38	0.35	− 0.03	0.95	0.45	0.41	− 0.03	0.87	0.66	0.24
Thursday	31	0.40	0.27	0.58	0.26	0.32	0.57	0.27	0.69	− 0.14	0.49	0.38	0.52
Friday	24	0.05	0.90	0.21	0.69	0.19	0.68	− 0.12	0.82	− 0.09	0.64	0.08	0.90
Season of sampling	191		0.65 ^f^		0.28 ^f^		0.64 ^f^		0.45 ^f^		0.35 ^f^		0.93 ^f^
Jan-March	59	0		0		0		0		0		0	
April-June	49	− 0.15	0.55	− 0.46	0.23	− 0.48	0.20	− 0.63	0.15	− 0.10	0.46	− 0.25	0.59
July-Sept	38	− 0.35	0.24	− 0.74	0.11	− 0.12	0.79	0.14	0.77	0.11	0.56	− 0.03	0.96
Oct-Dec	45	− 0.05	0.86	− 0.03	0.93	− 0.22	0.60	− 0.31	0.48	0.25	0.19	− 0.27	0.62
Duration of urine storage at room temperature (hours)	191		0.22 ^f^		0.04 ^f^		0.29 ^f^		0.54 ^f^		0.42 ^f^		0.33 ^f^
> 3	57	0		0		0		0		0		0	
3-4	51	− 0.33	0.24	− 0.71	0.09	0.67	0.13	− 0.17	0.67	0.03	0.87	− 0.16	0.74
4-5	32	0.14	0.72	0.23	0.70	0.15	0.78	− 0.62	0.21	0.23	0.28	− 1.1	0.12
> 5	51	− 0.10	0.81	− 0.31	0.58	− 0.09	0.88	− 0.07	0.91	0.27	0.19	− 0.20	0.75
*P* trend			0.91		0.71		0.70		0.94		0.16		0.78
Continuous ^e^		− 0.07	0.18	− 0.12	0.07	− 0.01	0.84	0.02	0.83	0.05	0.06	− 0.02	0.86

^a^ Linear regression models were corrected for over-representation of cases and adjusted for maternal age, bmi, parity, centre, education, occupation at the time of urine collection and urine sampling conditions.

^b^ Regression parameters are given for an increase by 1 in the sampling hour.

^c^ Regression parameters are given for an increase by 1 g/l in urinary creatinine concentration.

^d^ Regression parameters are given for an increase by 1 week.

^e^ Regression parameters are given for an increase by 1 hour.

^f^*P* of heterogeneity test

### Standardization of biomarker concentrations on sampling conditions

Table 4 shows the relative change in biomarker concentrations corrected for case – control sampling: after an additional standardization for sampling conditions, the strongest relative variations were observed for the concentrations of MCNP, for the metabolites of DEHP and MCOP (+80 %, +56 % and +44 %, respectively); median phenols concentrations varied between − 38 % for 2,5-DCP and +15 % for BPA. The correlation coefficients between log-transformed biomarker levels before and after standardization ranged between 0.88 for MBzP and 0.99 (P >  0.01) for triclosan (Table 4).

**Table 4:** Phthalates Metabolites and Phenols Urinary Concentrations Among Pregnant Women From Eden and Pélagie Cohorts, France, 2002-2006

		**Correction/standardization applied to the biomarker concentrations**																
		**No correction** **(Crude concentrations, μ g/l)^a^ (1)**			**Case – control sampling only ( μ g/l)^a^ (2)**			**Case – control sampling and creatinine** **( μ g/g Cr)^b^ (3)**			**Correction for case – control sampling and standardization for sampling conditions including creatinine ^c^ ( μ g/l)^a^ (4)**			**Correction for case – control sampling and standardization for sampling conditions excluding creatinine ^d^ ( μ g/l)^a^ (5)**			**Median change** **(5 – 2)^e^**	**Correlation standardized – unstandardized (5)(2) ^f^**
**Compound**	**n**	**Median**	**25^th^**	**75^th^**	**Median**	**25^th^**	**75^th^**	**Median**	**25^th^**	**75^th^**	**Median**	**25^th^**	**75^th^**	**Median**	**25^th^**	**75^th^**		
**Phthalate monoester metabolites**																		
MEHHP + MEOHP + MECPP + MEHP ( μ mol/l)	287	0.36	0.18	0.62	0.37	0.17	0.61	0.32	0.19	0.53	0.53	0.29	0.86	0.58	0.32	1.06	+ 56%	0.94
MiBP	287	50	29	97.5	45.9	30.2	84.0	45.7	28.0	71.9	59.2	39.0	101.5	64.7	41.2	119.3	+ 41%	0.94
MBP	287	57.5	30.5	104	48.1	28.9	86.7	48.5	27.6	75.5	57.5	35.9	86.4	58.1	34.3	105.6	+ 21%	0.96
MCCP	287	2.40	1.40	4.80	2.20	1.30	4.30	1.93	1.37	3.20	2.74	1.76	4.91	3.15	1.69	5.54	+ 43%	0.94
MBzP	287	18.5	10.4	40.3	17.7	9.3	33.5	16.0	8.6	28.0	20.4	9.7	42.9	21.7	9.6	57.7	+23%	0.88
MEP	287	112	49.0	236	110	53.3	215	106	54.4	198	103	58.7	196	105	53.9	240	− 5%	0.95
MCNP	287	1.90	1.10	3.40	1.70	1.10	3.00	1.59	1.15	2.51	2.89	2.05	5.00	3.06	1.89	5.63	+80%	0.95
MCOP	287	3.10	1.50	6.20	2.70	1.40	5.10	2.37	1.44	4.58	3.81	2.04	7.39	3.89	2.12	7.93	+44%	0.93
**Phenols**																		
2,4-DCP	191	0.90	0.50	1.60	0.90	0.50	1.50	0.78	0.51	1.48	0.78	0.47	1.51	0.77	0.49	1.59	− 14%	0.98
2,5-DCP	191	10.30	4.10	26.8	10.2	4.10	26.50	8.88	4.27	24.4	6.73	3.08	23.3	6.37	2.92	25.6	− 38%	0.95
Sum of Parabens ( μ mol/l)	191	0.90	0.28	2.36	0.86	0.27	2.45	0.81	0.29	2.15	0.86	0.30	2.31	0.95	0.29	2.32	+10%	0.98
BP3	191	1.60	0.60	4.10	1.70	0.70	4.40	1.63	0.64	4.19	1.13	0.59	3.87	1.30	0.54	4.04	− 24%	0.96
BPA	191	2.80	1.60	4.90	2.70	1.70	5.50	2.50	1.62	4.49	3.06	1.80	5.26	3.11	1.92	6.11	+15%	0.95
Triclosan	191	24.8	4.0	174	24.1	3.80	173	26.7	3.20	168.1	18.1	2.50	96.2	17.5	2.62	101.1	− 27%	0.99

We applied correction factors of 0.66 and 0.72 to the monoethyl phthalate (MEP) and monobenzyl phthalate (MBzP) concentrations, respectively, because the analytic standards used were of inadequate purity.

^a^ For metabolites of DEHP and sum of parabens, concentrations are reported in μ mol/l.

^b^ For metabolites of DEHP and sum of parabens, corrected concentrations are reported in μ mol/g Creatinine. Correction for creatinine was performed by dividing biomarker concentrations by creatinine concentration.

^c^ Results corrected for over-representation of cases according to Richardson et al. (2007) and standardized for all sampling conditions (including creatinine) using formula (1) in the methods section.

^d^ Results corrected for over-representation of cases according to Richardson et al. (2007) and standardized for sampling conditions (excluding creatinine) using formula (1) in the methods section.

^e^ Relative changes in the median concentration of urinary biomarkers between the estimates of column (5) and (2).

^f^ Coefficient of correlation (Pearson) between non-standardized biomarkers levels corrected for the case – control sampling and concentrations corrected for the case – control sampling and standardized for all sampling conditions excluding creatinine.

## Discussion

Within our population of pregnant women, the hour of urine collection (in the morning) was negatively associated with the concentration of most metabolites of phthalates (apart from MEP) and also of BPA (a result based on Eden cohort only). Standardization for sampling conditions modified the median concentrations by − 38 % for 2,5-DCP up to +80 % for MCNP, but standardized levels were relatively strongly correlated with unstandardized ones (correlation coefficients ranged between 0.88 for MBzP to 0.99 for triclosan).

Concentrations of some phthalate metabolites and of BPA decreased with increasing hour of collection in the morning. These changes may be due to exposure being more frequent at specific hours of the day (e.g. during the evening meal and less frequently in the night and early morning), and to the toxicokinetics of phthalates and phenols in each individual. In the case of Bisphenol A, for example, Teeguarden et al concluded that spot urine samples reflect exposure in the prior meal, or prior 4- to 6-hour period, but not during the whole 24-hour period preceding urine sampling [31]. Other studies in observational settings reported strong variations in biomarker urinary levels throughout the day [14,16]. We found no relation between gestational age at sampling and phthalates concentration. However, our study design had limitations to investigate this relation because we examined 2 distinct and relatively short periods of gestation for phthalates metabolites concentrations and only 1 for phenols concentrations, so that variability in sampling week was limited.

The decrease in the concentrations of MBP with the increasing duration of storage of urine samples at room temperature could be explained by microbial degradation or by irrecoverable adsorption of the monoesters metabolites to other urinary components or sediments [32]. The overlap between the 2 cohorts in the distributions of duration of storage was limited, so that the estimate of the influence of storage duration is mostly based on subjects from Eden cohort for durations shorter than 24 h, and on subjects from Pélagie cohort for durations of 24 h or more. The increasing concentration of BPA with increasing duration of storage at room temperature was unexpected; it might be due to a leakage of BPA from the plastic containers (or their caps) used to collect urine samples, as might happen if some women had used polycarbonate containers instead of the polyprolene containers planned for the study. Our analysis allowed to identify this potential issue and the statistical approach used attempted to correct for any resulting error.

The decrease in urinary concentrations of MBP, a metabolite of di-n-butyl phthalate (DnBP), observed from year 2005 onwards and the simultaneous increase in concentrations of MCOP, a metabolite of diisononyl phthalate (DiNP), could reflect changes in phthalates usage in Europe. DiNP is used as substitute to replace DEHP in many applications (ECPI, 2006): between 1999 and 2004, the proportion of DEHP to total phthalate usage decreased, and the proportion of DiNP and diisodecyl phthalate (DiDP) increased (ECPI, 2006). However, we did not observe a temporal decrease in DEHP metabolites.

Our results suggest that, like in other countries, French pregnant women are exposed to a range of non-persistent endocrine disruptors. MEP, MBP and MiBP were the phthalates found at the highest concentrations. The concentrations of these phthalate metabolites and of MCPP and the DEHP metabolites had the same magnitudes as those observed among pregnant women elsewhere [4,5,13,33,34]. MiBP concentrations reported in the USA among pregnant women [13,33] were lower than in our study (see Additional file 1: Figure S1). These geographical differences could be due to the fact that di-isobutyl phthalate, of which MiBP is a major metabolite, is used in Europe as a substitute of DnBP, banned by the European Union in personal care and cosmetic products [35].

Concerning phenols (see Additional file 1: Figure S2), after adjustment for creatinine, BPA concentrations were higher in our French population (median, 2.5  μ g/g) than those observed among pregnant women from Rotterdam (median, 1.6  μ g/g) [5], and from Cincinnati, Ohio (median at 16 gestational weeks, 1.7  μ g/g) [36]. Mean values were lower in Eden cohort (3.6  μ g/g) than in a study in Norway [4], where higher levels (creatinine-adjusted mean of 5.9  μ g/g) could be the consequence of a high consumption of canned fish and seafood [4].

The concentrations of biomarkers issued from biochemical assays cannot always be used in a straightforward way as an exposure variable in epidemiological studies [19,37] and may require additional modeling steps, just like for other exposure metrics. This can also probably apply to descriptive (biomonitoring) studies. Indeed, some degree of heterogeneity in sampling conditions is unavoidable in observational settings. The 2-step standardization method based on regression residuals that we proposed constitutes a way to reduce undesirable variability in biomarker urinary concentrations due to sampling conditions, and allows more relevant comparisons between subjects and possibly between studies. This source of variability can be seen as a source of measurement error in exposure, which may have impacts in studies of the association between biomarker levels and health, by impacting the regression models estimates in either direction [38] and/or confidence intervals. If we except the case of creatinine, which can be seen as a proxy for a sampling condition (time elapsed since the last void), and is very often corrected for in descriptive or etiologic studies, our report is to our knowledge one of the first attempts to limit variations in phthalate and phenols biomarker levels due to variations in urine sampling conditions in a descriptive setting using a statistical approach.

In a further step, we suggest to use the standardized biomarker concentration to characterize the relation between biomarker levels and specific health outcomes assessed in the same population [24]. Further developments of our approach that may be useful for such etiological studies would be to acknowledge for the variability in the regression coefficients corresponding to the effect of sampling conditions on biomarkers estimated in the measurement model (Eq. A.1, see Additional file 2 statistical appendix). In particular, regression models in which the standardized concentrations are used as covariates should take this variability into account. Incidentally, it can be noted that using unstandardized (raw) levels in models not accounting for measurement error due to variability in sampling conditions can also impact on variance estimates and on bias; we believe that an approach like ours, aiming at making sources of measurement error explicit and at correcting for them, is a step in the good direction. We chose to standardize each biomarker level on all sampling conditions simultaneously, but in future studies authors may prefer to standardize only for those sampling conditions that turn out to be associated with the considered biomarker with a p value below a given level (say, p >  0.2).

As an alternative to using standardized biomarker levels, some authors include sampling characteristics as covariates in regression models describing associations between biomarker levels and health outcome; this 1-step approach may not allow efficiently standardizing sampling conditions, as the parameter associated with sampling conditions will reflect the association between the sampling condition and the health outcome, and not with the biomarker level [19]; our 2-step approach allows to separately consider the influence of sampling conditions on biomarker levels in a first step and to correct for it in a second step that does not consider the health outcome; in a final step, the association between standardized biomarker concentrations and the health outcome can be characterized. There is a vast body of literature on how to handle and try to correct measurement error in covariates or health outcomes [39]. However, it is focused on situations in which there is some knowledge either on the standard errors attached to the error-prone variables or on the misclassification rate, on situations in which validation data in which both true and error-prone variables have been assessed in a sub-populations, or in which instrumental variables are available. These situations do not correspond to ours, in which we do know and measure some factors causing measurement error (the sampling conditions), and empirically estimate the influence of these factors on the mismeasured concentrations.

The impact of sampling conditions on biomarker levels was empirically estimated based on the association observed in our data. An alternative would be to use a toxicokinetic model; however such models are not currently available for most of the studied metabolites [40,41], in particular for pregnant human subjects; only limited data on the half-life or other toxicokinetic parameters of the studied compounds are available, in populations distinct from pregnant women, who are different from non-pregnant women in terms of metabolism for specific xenobiotics [42]. The lack of repeated assessment and of information on timing of exposure did not allow to develop such toxicokinetic modeling within our population.

Our two-step approach is to our knowledge original although it follows a logic previously used in some biomarkers studies [19], and also in other areas of the epidemiologic and clinical literature, for example in studies of lung function, in which results of a lung function test (e.g., FEV1) are standardized on gender and age, to limit the impact of these (nuisance) factors.

Each pregnant woman provided only a single urine sample, which probably limited the accuracy of our estimates of the influence of sampling conditions. In practice, many studies in the general population rely on a single urine sample, and standardizing for sampling conditions should also be attempted in this setting. We assumed that adjustment for individual characteristics such as age, occupation or smoking, made women with different sampling conditions more comparable. However, this approach might be limited by the existence of unmeasured lifestyle or occupational factors simultaneously associated with exposure and sampling conditions. For instance, if women who collected a urine sample early in the morning used more phthalate-containing cosmetics than those who provided a urine sample later in the morning, we might attribute to variations in sampling hour differences actually due to real exposure contrasts. Time since last exposure (and amount of exposure) are also parameters likely to influence biomarker levels. These were not available in our study; their assessment is challenging in observational studies focused on compounds with several sources and whose presence in consumers’ products is not known by study participants. Moreover, time since last exposure is likely to be shorter for subjects frequently exposed to these compounds (and hence also possibly more highly exposed to these compounds), so that standardization for time since last exposure might artificially decrease the between-subject contrasts in exposure.

The efficiency of our approach may differ between compounds. In the case of standardization for sampling hour, for example, the approach is more likely to be efficient for compounds in which biomarker levels in urine follow a similar temporal pattern throughout the day for most participants; such a situation is close to what has been described for MEP [14]. For other compounds for which temporal patterns strongly differ between participants, as has been described for mono-(2-ethyl-5-hydroxyhexyl) phthalate [14], our approach is likely to be less efficient; in such cases, there may be no efficient statistical alternative to collecting several urine samples per day or 24-hour urine samples [14], at least in a sub-population, after which measurement error models [39,43] or toxicokinetic models (if available) could be used. Similarly, if there is no consistent pattern of variation in exposure levels throughout the week, as seemed to be the case in our population for most compounds, our approach is unlikely to correct for daily variations in exposure and thus to make biomarker levels more representative of the weekly exposure average.

For the above-mentioned reasons, some error in our estimates of the influence of sampling conditions on biomarker concentration is expected, so that we cannot exclude that the standardized concentrations sometimes entail more bias than the original measure [44]. Consequently, studies on exposure-response relations using an approach such as ours should also report the association between the uncorrected biomarker concentrations and the health outcome, in addition to the association relying on standardized biomarker concentrations [24]. Furthermore, information on sampling conditions such as those considered here (in addition, whenever relevant, to batch number, assay date, and information on any deviation from the planned protocol) should be collected for all study subjects so that their possible impact can be characterized and if required corrected for.

## Conclusions

In conclusion, hour of sampling was associated with the urinary concentrations of select phthalate metabolites and phenols. This confirms the relevance for studies aiming to characterize the health effect of compounds with a short half-life such as phthalates and phenols to rely on *repeated* biomarker assays. Our approach used to standardize concentrations of biomarkers in urine specimens collected under varying conditions (e.g., time of day) could be relevant for future studies aiming at describing the urinary concentrations of biomarkers, or their influence on human health outcomes.

## List of Abbreviations

DEHP: Di(2-ethylhexyl) phthalate; MEHHP: Mono-(2-ethyl-5-hydroxyhexyl) phthalate; MEOHP: Mono-(2-ethyl-5-oxohexyl) phthalate; MECPP: Mono-(2-ethyl-5-carboxypentyl) phthalate; MEHP: Mono-(2-ethylhexyl) phthalate; DiBP: Di-iso-butyl phthalate; MiBP: Monoisobutyl phthalate; DnBP: Di-n-butyl phthalate; MBP: Mono-n-butyl phthalate; DOP: Di-n-octyl phthalate; MCPP: Mono-3-carboxypropyl phthalate; BzBP: Benzylbutyl phthalate; MBzP: Monobenzyl phthalate; DEP: Diethylphthalate; MEP: Monoethyl phthalate; DiDP: Diisodecyl phthalate; MCNP: Mono-(2,7-dimethyl-7-carboxyheptyl) phthalate; DiNP: Diisononyl phthalate; MCOP: Mono-(2,6-dimethyl-6-carboxyhexyl) phthalate; 2,4-DCP: 2,4-dichlorophenol: 2,5-DCP: 2,5-dichlorophenol; BP: Butyl paraben; EP: Ethyl paraben; MP: Methyl paraben; PP: Propyl paraben; BP3: Benzophenone 3; BPA: Bisphenol A; TCS: Triclosan.

## Competing interests

The authors declare that they have no competing financial interests.

## Authors’ contributions

M.M. and R.S. designed the analysis plan, analyzed data and wrote the paper. R.S. suggested the 2-step standardization method based on regression residuals. C.Ph. contributed to the data analysis. R.S., S.C. and C.C. designed the study and directed its implementation. S.C. supervised the implementation of Pélagie cohort, and M.A.C. supervised the implementation of Eden cohort. A.M.C., X.Y. and M.J.S. were involved in issues related to exposure assessment, supervised and performed measurement of phthalates metabolites and phenols concentrations. A.M.C., C.Ph., C.Pe. and all other authors discussed the results and implications and commented on the manuscript at all stages. All authors read and approved the final manuscript.

## Supplementary Material

Additional file 1 Statistical appendix. Click here for file

Additional file 2 **Table S1**. Limits of Detection of Urinary Phthalates Metabolites and Phenols and Detection Frequency Among FrenchPregnant Women from Eden and Pelagie Cohorts, 2002 – 2006. **Table S2**. Correlation coefficients between Phthalates Monoesters Metabolites and Phenols Crude Urinary Concentrations Among French Pregnant Women from Eden and Pelagie Cohorts, 2002 – 2006.**Table S3**. Adjusted Association Between Log-Transformed Phthalate Monoester Metabolites Urinary Concentrations and Characteristics of French Pregnant Women From Eden and Pélagie Cohorts, 2002 – 2006. Linear Regression Models Were Corrected for Over-Representation of Cases and Adjusted for Maternal age, BMI, Parity, Centre, Education, Occupation and Urine Sampling Conditions. **Table S4**. Adjusted Association Between Log-Transformed Phenol Urinary Concentrations and Characteristics of French Pregnant Women from Eden and Pelagie Cohorts, 2002 – 2006. Linear Regression Models Were Corrected for Over-Representation of Cases and Adjusted for Maternal age, BMI, Parity, Centre, Education, Occupation and Urine Sampling Conditions. **Figure S1**. Median Values of Urinary Phthalate Metabolites ( μ g/l) in Selected Publications Among Pregnant Women. **Figure S2**. Median Values of Urinary Phenols ( μ g/l) in Selected Publications Among Pregnant Women. Click here for file
