# Supplementary material for: Correcting for the influence of sampling conditions on biomarkers of exposure to phenols and phthalates: a 2-step standardization method based on regression residuals
Source: Environ Health. 2012 Apr 26;11:29. doi: 10.1186/1476-069X-11-29 (PMC3533777; doi:10.1186/1476-069X-11-29)
Supplement: Additional file 1 — Statistical appendix. [file 1476-069X-11-29-S1.pdf]

Correcting for the influence of sampling conditions on biomarkers of exposure to phenols and phthalates: a 2-step standardization method based on regression residuals.

Marion Mortamais[1, 2], Cécile Chevrier[3], Claire Philippat[1, 4], Claire Petit[3], Antonia M. Calafat[5], Xiaoyun Ye[5], Manori J. Silva[5], Christian Brambilla[6], Marinus J.C. Eijkemans[7], Marie-Aline Charles[8], Sylvaine Cordier[3], Rémy Slama[1, 4*].

[1]Team of Environmental Epidemiology applied to Reproduction and Respiratory Health, Inserm, Institut Albert Bonniot (U823), Grenoble, France
[2] Inserm, U1061, Montpellier, France
[3] Inserm, U625, Rennes, France
[4] Grenoble University, Institut Albert Bonniot (U823), Grenoble, France
[5] Centers for Disease Control and Prevention, Atlanta, GA, USA
[6] Inserm and Grenoble University, Institut Albert Bonniot (U823), Molecular Basis of lung cancer progression, Grenoble, France
[7] Julius Center for Health Sciences and Primary Care, UMC Utrecht, The Netherlands
[8] Inserm and INED joint research group, PARIS, and Inserm, U1018, CESP, Villejuif, France

Statistical appendix

In order to standardize the biomarker concentration on sampling conditions (including hour of sampling, delay between urine collection and freezing), we take away from the observed biomarker concentration a value depending on how much the sampling conditions for subject i differ from the *standard* sampling conditions, i.e. those that should have been observed for the whole population in ideal conditions. This 2-step standardization method based on regression residuals is described below.

For each biomarker, we first fit a *measurement model* (Eq. A.1), corresponding to a linear regression model of ln-transformed biomarker concentration ln([Conc]) = Y, including as covariates all sampling conditions and potential confounders:

$$(Y^i)^{measured} = \alpha + \Sigma_j[\beta_{samp\ cond\ j} \text{ x } X^i_j\ ] + \Sigma_k[\gamma_k \text{ x } Z^i_k\ ] + \varepsilon^i \qquad \text{(Eq. A.1)}$$

Where $(Y^i)^{measured}$ is the measured concentration in subject i, $\beta_{samp\ cond\ j}$ is the regression parameter quantifying the effect of sampling condition X_j on the biomarker's concentration, and Z_k correspond to the potential confounders (age, socio-economic status, smoking, etc.). The model's residuals (ε^i) correspond to the variability in Y^i not explained by sampling characteristics and potential confounders; ε^i therefore includes the 'informative' part of the biomarker levels, in particular that due to variations in exposure.

In a second step, the standardized concentration $(Y^i)^{standardized}$ is estimated as:

$$(Y^i)^{standardized} = (Y^i)^{measured} - \Sigma_j[\beta_{samp\ cond\ j} \text{ x } (X_j^i\text{-}X_j^{std})] \qquad \text{(Eq. A.2)}$$

where X_j^{std} corresponds to the *standard* value for sampling condition j (*e.g.*, 7:30 AM, in the case of sampling hour), i.e. to the sampling condition that would have been observed if the sampling protocol had been strictly followed for all study participants.

Equation (A.2) can be justified the following way:

We start by writing that the expected ln-transformed biomarker concentration for subject i if sampling conditions correspond to the standard ones (and if potential confounders Z have the values corresponding to those observed for each subject i) is:

$$(Y^i)^{standardized} = \Sigma_j[\beta_{samp\ cond\ j} \text{ x } X_j^{std}] + \Sigma_k[\gamma_k \text{ x } Z^i_k\ ] + \varepsilon^i \qquad \text{(Eq. A.3)}$$

Equivalently to (Eq. A.1), one can write:

$$\varepsilon^i = (Y^i)^{measured} - \Sigma_j[\beta_{samp\ cond\ j} \times X^i_j] - \Sigma_k[\gamma_k \times Z^i_k] \qquad \text{(Eq. A.4)}$$

We now assume that the residuals ε of equations (A.1) and (A.3) are identical. This will be the case if these residuals are uncorrelated to the covariates X and potential confounders Z, and if the effect measure of X on Y is not modified by Z. With these assumptions, we now replace ε^i in Eq. A.3 by its expression Eq A.4, which gives:

$$(Y^i)^{standardized} = \Sigma_j[\beta_{samp\ cond\ j} \times X_j^{std}] + \Sigma_k[\gamma_k \times Z^i_k] + (Y^i)^{measured} - \Sigma_j[\beta_{samp\ cond\ j} \times X_j^i] - \Sigma_k[\gamma_k \times Z^i_k]$$

$$= (Y^i)^{measured} - \Sigma_j[\beta_{samp\ cond\ j} \times (X_j^i - X_j^{std})]$$

which corresponds to equation A.2.

One can note that the standardized biomarker concentration corresponds to the measured one for subjects for whom samples were collected according to the standard sampling conditions ($X^i=X^{std}$), and that the corrective factor applied to the measured concentration becomes larger (in absolute value) as X^i moves away from X^{std}, which corresponds to what one would intuitively expect.

In a third step (not presented in this article), one can use the standardized biomarker concentration to assess the relation between biomarker levels and specific health outcomes assessed in the same population. In doing so, several issues need to be kept in mind:

1) The approach outlined here yields an estimate of the standardized ln-transformed concentration; caution is required if one wishes to work on concentration itself instead of ln-transformed concentration; in theory, the estimated standardized (untransformed)

concentration may not correspond to the exponential of the estimated standardized ln-transformed concentration (see e.g., [35], p.159-160);

2) The estimated standardized ln-transformed concentrations will have an additional source of variability, corresponding to the variability in the estimated regression coefficients corresponding to the effect of sampling conditions in the measurement model Eq. A.1; this variability will also vary with the observed value of the sampling conditions X. Regression models in which the standardized concentrations are used as covariates should take this change in variance into account.
