# Supplementary material for: Correcting for the influence of sampling conditions on biomarkers of exposure to phenols and phthalates: a 2-step standardization method based on regression residuals
Source: Environ Health. 2012 Apr 26;11:29. doi: 10.1186/1476-069X-11-29 (PMC3533777; doi:10.1186/1476-069X-11-29)
Supplement: Additional file 2 — Table S1. Limits of Detection of Urinary Phthalates Metabolites and Phenols and Detection Frequency Among FrenchPregnant Women from Eden and Pelagie Cohorts, 2002 – 2006. Table S2. Correlation coefficients between Phthalates Monoesters Metabolites and Phenols Crude Urinary Concentrations Among French Pregnant Women from Eden and Pelagie Cohorts, 2002 – 2006.Table S3. Adjusted Association Between Log-Transformed Phthalate Monoester Metabolites Urinary Concentrations and Characteristics of French Pregnant Women From Eden and Pélagie Cohorts, 2002 – 2006. Linear Regression Models Were Corrected for Over-Representation of Cases and Adjusted for Maternal age, BMI, Parity, Centre, Education, Occupation and Urine Sampling Conditions. Table S4. Adjusted Association Between Log-Transformed Phenol Urinary Concentrations and Characteristics of French Pregnant Women from Eden and Pelagie Cohorts, 2002 – 2006. Linear Regression Models Were Corrected for Over-Representation of Cases and Adjusted for Maternal age, BMI, Parity, Centre, Education, Occupation and Urine Sampling Conditions. Figure S1. Median Values of Urinary Phthalate Metabolites ( μ g/l) in Selected Publications Among Pregnant Women. Figure S2. Median Values of Urinary Phenols ( μ g/l) in Selected Publications Among Pregnant Women. [file 1476-069X-11-29-S2.pdf]

Correcting for the influence of sampling conditions on biomarkers of exposure to phenols and phthalates: a 2-step standardization method based on regression residuals.

Marion Mortamais[1, 2], Cécile Chevrier[3], Claire Philippat[1, 4], Claire Petit[3], Antonia M. Calafat[5], Xiaoyun Ye[5], Manori J. Silva[5], Christian Brambilla[6], Marinus J.C. Eijkemans[7], Marie-Aline Charles[8], Sylvaine Cordier[3], Rémy Slama[1, 4*].

[1]Team of Environmental Epidemiology applied to Reproduction and Respiratory Health, Inserm, Institut Albert Bonniot (U823), Grenoble, France

[2] Inserm, U1061, Montpellier, France

[3] Inserm, U625, Rennes, France

[4] Grenoble University, Institut Albert Bonniot (U823), Grenoble, France

[5] Centers for Disease Control and Prevention, Atlanta, GA, USA

[6] Inserm and Grenoble University, Institut Albert Bonniot (U823), Molecular Basis of lung cancer progression, Grenoble, France

[7] Julius Center for Health Sciences and Primary Care, UMC Utrecht, The Netherlands

[8] Inserm and INED joint research group, PARIS, and Inserm, U1018, CESP, Villejuif, France

***Corresponding author**:

Telephone: +33 476 54 94 02

Fax: +33 476 54 94 14

E-mail: remy.slama@ujf-grenoble.fr

Table 1, Supplemental Material. Limits of Detection of Urinary Phthalates Metabolites and Phenols and Detection Frequency Among French Pregnant Women from Eden and Pelagie Cohorts, 2002-2006. 3

Table 2, Supplemental Material. Correlation coefficients between Phthalates Monoesters Metabolites and Phenols Crude Urinary Concentrations Among French Pregnant Women from Eden and Pelagie Cohorts, 2002-2006. 4

Table 3, Supplemental Material. Adjusted Association Between Log-Transformed Phthalate Monoester Metabolites Urinary Concentrations and Characteristics of French Pregnant Women From Eden and Pélagie Cohorts, 2002-2006. Linear Regression Models Were Corrected for Over-Representation of Cases and Adjusted for Maternal age, BMI, Parity, Centre, Education, Occupation and Urine Sampling Conditions 5

Table 4, Supplemental Material. Adjusted Association Between Log-Transformed Phenol Urinary Concentrations and Characteristics of French Pregnant Women from Eden and Pelagie Cohorts, 2002-2006. Linear Regression Models Were Corrected for Over-Representation of Cases and Adjusted for Maternal age, BMI, Parity, Centre, Education, Occupation and Urine Sampling Conditions.. 7

Figure 1, Supplemental Material. Median Values of Urinary Phthalate Metabolites (µg/l) in Selected Publications Among Pregnant Women. 9

Figure 2, Supplemental Material. Median Values of Urinary Phenols (µg/l) in Selected Publications Among Pregnant Women. 10

Table 1, Supplemental Material. Limits of Detection of Urinary Phthalates Metabolites and Phenols and Detection Frequency Among French Pregnant Women from Eden and Pélagie Cohorts, 2002-2006.

Parent compound	Metabolite	n	LOD (µg/l)	Frequency of detection (%)
**Esters of phthalates**				
DEHP				
	MEHHP	287	0.7	100
	MEOHP	287	0.7	99.7
	MECPP	287	0.6	100
	MEHP	287	1.2	92
DiBP	MiBP	287	0.3	100
DnBP	MBP	287	0.6	100
DOP	MCPP	287	0.2	98.6
BzBP	MBzP	287	0.3	100
DEP	MEP	287	0.8	100
DiDP	MCNP	287	0.6	92.3
DiNP	MCOP	287	0.7	92.3
**Phenols**				
2,4-DCP		191	0.2	96.3
2,5-DCP		191	0.2	99.5
BP		191	0.2	80.1
EP		191	1.0	68.1
MP		191	1.0	100
PP		191	0.2	96.9
BP3		191	0.4	80.1
BPA		191	0.4	99
Triclosan		191	2.3	84.8

The frequency of detection has been corrected by the over-sampling of cases due to the case-control design. Concentrations of monoethyl phthalate (MEP) and monobenzyl phthalate (MBzP) had been multiplied by 0.66 and 0.72, respectively, because the analytic standards used were of inadequate purity.

Table 2, Supplemental Material. Correlation coefficients between Phthalates Monoesters Metabolites and Phenols Log Transformed Crude Urinary Concentrations Among French Pregnant Women From Eden and Pélagie Cohorts, 2002-2006.

Compound	MEHHP+MEOHP+ MECPP+MEHP	MiBP	MBP	MCPP	MBzP	MEP	MCNP	MCOP	2,4-DCP	2,5-DCP	Sum of Parabens	BP3	BPA	TCS
MEHHP+MEOHP+MECPP +MEHP	1.00													
MiBP	0.56[1]	1.00												
MBP	0.52[1]	0.49[1]	1.00											
MCPP	0.57[1]	0.48[1]	0.76[1]	1.00										
MBzP	0.58[1]	0.61[1]	0.49[1]	0.47[1]	1.00									
MEP	0.31[1]	0.26[1]	0.29[1]	0.24[1]	0.28[1]	1.00								
MCNP	0.42[1]	0.40[1]	0.3[1]5	0.54[1]	0.40[1]	0.17[1]	1.00							
MCOP	0.50[1]	0.43[1]	0.37[1]	0.55[1]	0.45[1]	0.32[1]	0.58[1]	1.00						
2.4-DCP	0.20[1]	0.19[1]	0.14	0.08	0.12	0.21[1]	0.18[1]	0.24[1]	1.00					
2.5-DCP	0.16[1]	0.22[1]	0.07	0.04	0.09	0.10	0.16[1]	0.19[1]	0.87[1]	1.00				
Sum of Parabens	-0.03	0.08	0.06	0.08	0.01	0.24[1]	0.12	0.05	0.14	0.09	1.00			
BP3	0.09	0.13	0.06	0.03	0.12	0.24[1]	0.08	0.15[1]	0.10	0.13	0.28[1]	1.00		
BPA	0.46[1]	0.48[1]	0.41[1]	0.50[1]	0.43[1]	0.28[1]	0.35[1]	0.44[1]	0.05	-0.004	0.02	0.14	1.00	
Triclosan	0.12	-0.006	0.07	0.001	0.01	0.26[1]	0.04	0.08	0.39[1]	0.13	0.10	0.13	-0.07	1.00

[1] $p<0.05$.

Table 3, Supplemental Material. Adjusted Association Between Log-Transformed Phthalate Monoester Metabolites Urinary Concentrations and Characteristics of French Pregnant Women From Eden and Pélagie Cohorts, 2002-2006. Linear Regression Models Were Corrected for Over-Representation of Cases and Adjusted for Maternal age, BMI, Parity, Centre, Education, Occupation and Urine Sampling Conditions.

		MEHHP+MEOHP +MECPP+MEHP		MiBP		MBP		MCPP		MBzP		MEP		MCNP		MCOP	
Characteristics of participants	n	β	*P*	β	*P*	β	*P*	β	*P*	β	*P*	β	*P*	β	*P*	β	*P*
Date of sampling	287		0.52[g]		<0.01[g]		<0.01[g]		0.14[g]		0.32[g]		0.73[g]		0.08[g]		<0.01[g]
< 1 Jan 2004	72	0		0		0		0		0		0		0		0	
1 Jan 2004 to 27 May 2004	69	-0.01	0.94	0.03	0.89	0.01	0.95	-0.02	0.91	-0.03	0.90	0.17	0.43	0.006	0.98	0.17	0.35
28 May 2004 to 23 Feb 2005	73	0.03	0.93	0.20	0.27	-0.16	0.45	-0.22	0.27	-0.13	0.60	-0.06	0.73	0.12	0.52	0.35	0.05
≥ 24 Feb 2005	73	-0.21	0.33	0.10	0.66	-0.65	<0.01	-0.45	0.06	-0.42	0.11	-0.02	0.92	-0.32	0.12	0.73	<0.01
P trend			0.54		0.45		<0.01		0.04		0.13		0.68		0.29		<0.01
Continuous [a]		-0.12	0.19	0.04	0.68	-0.24	0.02	-0.19	0.04	-0.18	0.09	-0.02	0.84	-0.10	0.25	0.31	<0.01
Maternal age (years)	287		0.28[g]		0.24[g]		0.01[g]		0.07[g]		0.05[g]		0.40[g]		0.32[g]		0.17[g]
< 25	44	0		0		0		0		0		0		0		0	
≥ 25 to 29	113	-0.28	0.17	-0.01	0.96	0.76	<0.01	0.27	0.23	0.43	0.08	0.32	0.12	-0.13	0.57	0.06	0.74
≥ 30 to 34	88	-0.37	0.06	-0.25	0.24	0.40	0.08	-0.12	0.61	0.0009	0.99	0.37	0.12	-0.26	0.29	-0.24	0.24
> 35	42	-0.41	0.12	-0.36	0.15	0.28	0.29	-0.02	0.94	0.01	0.96	0.35	0.24	-0.40	0.12	-0.30	0.26
P trend			0.13		0.05		0.54		0.19		0.22		0.36		0.07		0.05
Continuous [a]		-0.01	0.42	-0.03	0.02	-0.01	0.51	-0.01	0.43	-0.03	0.14	0.01	0.54	-0.02	0.19	-0.02	0.31
BMI [b](kg/m²)	287		0.08[g]		0.11[g]		0.03[g]		0.42[g]		0.55[g]		0.50[g]		0.71[g]		0.72[g]
< 18.5	29	0		0		0		0		0		0		0		0	
≥ 18.5 to 24.99	185	-0.30	0.16	-0.19	0.33	-0.65	0.01	-0.33	0.15	0.08	0.73	-0.36	0.13	0.03	0.89	-0.11	0.63
≥ 25 to 29.99	47	-0.50	0.02	-0.34	0.09	-0.76	<0.01	-0.40	0.12	-0.07	0.79	-0.30	0.30	0.07	0.78	0.07	0.79
> 30	26	-0.21	0.44	0.28	0.39	-0.64	0.05	-0.40	0.14	0.40	0.25	-0.27	0.39	0.36	0.28	0.06	0.85
P trend			0.17		0.77		0.05		0.16		0.62		0.64		0.36		0.42
Continuous [c]		-0.02	0.49	0.0005	0.99	-0.02	0.60	0.02	0.44	0.05	0.22	-0.03	0.48	-0.0002	0.99	0.04	0.20
Parity (previous livebirths)	287		0.27[g]		0.48[g]		0.54[g]		0.53[g]		0.36[g]		0.06[g]		0.09[g]		0.26[g]
0	115	0		0		0		0		0		0		0		0	
1	114	0.28	0.11	0.05	0.74	0.03	0.86	0.15	0.27	0.21	0.20	-0.40	0.02	-0.03	0.84	0.14	0.32
≥ 2	58	0.13	0.54	0.22	0.23	0.21	0.29	0.12	0.52	0.24	0.30	-0.10	0.68	0.42	0.06	0.35	0.11
P trend			0.26		0.30		0.40		0.34		0.19		0.24		0.18		0.10
Continuous [d]		0.10	0.27	0.09	0.21	0.08	0.31	0.06	0.39	0.11	0.23	-0.06	0.63	0.16	0.07	0.15	0.08
Centre	287		0.86[g]		0.42[g]		0.23[g]		0.36[g]		0.87[g]		0.94[g]		0.77[g]		0.66[g]
Poitiers	91	0		0		0		0		0		0		0		0	
Nancy	100	-0.11	0.58	0.21	0.27	0.26	0.18	-0.02	0.92	-0.11	0.61	0.03	0.92	0.13	0.52	-0.16	0.37
Pélagie	96	-0.26	0.87	-0.88	0.58	2.8	0.13	2.1	0.18	-0.49	0.80	0.72	0.72	0.69	0.71	0.02	0.99
Maternal education	287		0.45[g]		0.02[g]		0.59[g]		0.57[g]		<0.01[g]		0.91[g]		0.98[g]		0.05[g]
Primary education	5	-0.16	0.61	0.19	0.58	0.50	0.12	0.33	0.32	0.10	0.79	0.12	0.86	-0.05	0.87	-0.32	0.28
< High school	75	0		0		0		0		0		0		0		0	

High school	56	-0.30	0.09	-0.38	0.04	0.03	0.90	-0.18	0.29	-0.51	0.03	-0.18	0.37	-0.09	0.64	-0.31	0.11
High school + 2 years	62	-0.003	0.99	-0.57	<0.01	0.07	0.73	-0.17	0.38	-0.75	<0.01	-0.08	0.74	-0.13	0.57	-0.53	<0.01
≥ High school + 3 years	89	0.001	0.99	-0.18	0.29	-0.001	0.99	-0.05	0.75	-0.40	0.04	-0.15	0.51	-0.11	0.54	-0.36	0.05
Occupation [e]	287																
No	70	0		0		0		0		0		0		0		0	
Yes	217	0.12	0.49	0.22	0.30	-0.29	0.14	-0.13	0.44	0.06	0.76	-0.03	0.90	0.35	0.07	0.23	0.18
Active smoking	287		0.34 [g]		0.59 [g]		0.76 [g]		0.07 [g]		0.87 [g]		0.13 [g]		0.22 [g]		0.92 [g]
0	240	0		0		0		0		0		0		0		0	
1-5 cigarettes/day	30	0.03	0.92	-0.18	0.33	0.03	0.92	-0.16	0.47	-0.10	0.66	0.11	0.58	0.30	0.29	-0.12	0.53
6-10 cigarettes/day	12	-0.13	0.60	-0.13	0.70	0.02	0.95	0.32	0.08	-0.10	0.82	-0.29	0.15	1.3	0.08	6e-07	1.0
≥ 10 cigarettes/day	5	0.71	0.08	0.23	0.42	0.45	0.28	0.50	0.07	0.29	0.50	1.4	0.08	-0.13	0.72	-0.10	0.73
P trend			0.73		0.54		0.67		0.55		0.82		0.43		0.07		0.60
Continuous [f]		0.01	0.70	-0.01	0.63	0.02	0.51	0.01	0.71	0.002	0.95	0.03	0.30	0.07	0.11	-0.005	0.83

[a] Regression parameters given for an increase by 1 year.
[b] Before pregnancy.
[c] Regression parameters are given for an increase by 1 kg/ m² in body mass index.
[d] Regression parameters are given for each additional child.
[e] Occupation at time of collection of urine sample (yes/no).
[f] Regression parameters are given for an increase by one cigarette/day.
[g] *P* of heterogeneity test.

Table 4, Supplemental Material. Adjusted Association Between Log-Transformed Phenol Urinary Concentrations and Characteristics of French Pregnant Women From Eden Cohort, 2002-2006. Linear Regression Models Were Corrected for Over-Representation of Cases and Adjusted for Maternal Age, BMI, Parity, Centre, Education, Occupation at the Time of Urine Collection and Urine Sampling Conditions.

		2,4-DCP		2,5-DCP		Sum of Parabens		BP3		BPA		TCS	
Characteristics of participants	n	β	*P*	β	*P*	β	*P*	β	*P*	β	*P*	β	*P*
Date of sampling	191		0.07[g]		0.04[g]		0.62[g]		0.19[g]		0.22[g]		0.26[g]
< 1 Jan 2004	19	0		0		0		0		0		0	
1 Jan 2004 to 27 May 2004	45	-0.04	0.91	-0.12	0.81	0.68	0.19	1.1	0.08	0.27	0.36	-0.18	0.80
28 May 2004 to 23 Feb 2005	60	0.64	0.07	0.95	0.05	0.37	0.39	0.67	0.14	-0.04	0.88	0.37	0.59
≥ 24 Feb 2005	67	0.06	0.85	0.05	0.91	0.37	0.41	1.2	0.03	0.30	0.32	-0.54	0.41
P trend			0.57		0.57		0.95		0.19		0.52		0.42
Continuous [a]		0.08	0.60	0.19	0.39	-0.02	0.93	0.35	0.11	0.08	0.44	-0.29	0.32
Maternal age (years)	191		0.37[g]		0.70[g]		0.31[g]		0.02[g]		0.03[g]		0.12[g]
< 25	35	0		0		0		0		0		0	
≥ 25 to 29	71	0.34	0.23	0.28	0.52	-0.15	0.71	-0.85	0.08	-0.19	0.30	0.76	0.16
≥ 30 to 34	57	0.59	0.10	0.63	0.28	0.16	0.71	-0.29	0.62	-0.54	<0.01	1.3	0.02
> 35	28	0.25	0.46	0.24	0.64	0.69	0.22	0.59	0.44	-0.33	0.23	0.78	0.24
P trend			0.39		0.48		0.13		0.19		0.07		0.16
Continuous [a]		0.02	0.37	0.03	0.39	0.03	0.36	0.08	0.12	-0.01	0.46	0.04	0.32
BMI[b] (kg/m²)	191		0.22[g]		0.91[g]		0.43[g]		0.09[g]		0.57[g]		0.17[g]
< 18.5	19	0		0		0		0		0		0	
≥ 18.5 to 24.99	118	-0.40	0.22	-0.25	0.59	0.23	0.59	0.65	0.04	-0.11	0.49	-0.42	0.47
≥ 25 to 29.99	34	-0.05	0.89	-0.04	0.93	-0.45	0.40	0.92	0.05	-0.23	0.28	0.61	0.34
> 30	20	-0.03	0.95	-0.14	0.82	0.21	0.71	0.95	0.11	-0.26	0.23	-0.39	0.62
P trend			0.45		0.95		0.54		0.15		0.27		0.56
Continuous [c]		-0.10	0.03	-0.06	0.32	0.11	0.11	-0.03	0.74	-0.002	0.93	-0.12	0.17
Parity (previous livebirths)	191		0.89[g]		0.95[g]		0.02[g]		0.02[g]		<0.01[g]		0.37[g]
0	72	0		0		0		0		0		0	
1	75	0.12	0.64	0.08	0.83	-0.83	0.02	-0.92	0.01	0.20	0.19	-0.40	0.34
≥ 2	44	0.06	0.84	0.13	0.75	-0.97	0.01	-0.87	0.04	0.58	<0.01	-0.74	0.17
P trend			0.78		0.74		<0.01		0.02		<0.01		0.16
Continuous [d]		-0.06	0.56	-0.004	0.98	-0.33	0.05	-0.18	0.27	0.18	<0.01	-0.46	0.01
Centre	191												
Poitiers	91	0		0		0		0		0		0	
Nancy	100	0.49	0.08	0.65	0.11	0.12	0.73	-0.12	0.78	-0.39	<0.01	0.08	0.86
Maternal education	191		0.25[g]		0.31[g]		0.08[g]		0.57[g]		0.32[g]		0.29[g]
Primary education	5	-0.52	0.23	-0.72	0.28	1.2	0.03	1.0	0.37	-0.55	0.12	-1.5	0.05

< High school	56	0		0		0		0		0		0	
High school	37	-0.43	0.11	-0.64	0.10	0.14	0.72	-0.37	0.34	-0.06	0.75	-0.003	0.99
High school + 2 years	39	-0.53	0.07	-0.72	0.12	0.63	0.12	0.37	0.55	0.20	0.24	-0.009	0.99
≥ High school + 3 years	54	-0.24	0.37	-0.31	0.45	0.95	0.04	0.25	0.62	-0.02	0.92	0.25	0.61
Occupation [e]	191												
No	58	0		0		0		0		0		0	
Yes	133	0.09	0.67	-0.03	0.91	-0.004	0.99	0.09	0.80	-0.11	0.56	-0.12	0.76
Active smoking	191		0.43 [g]		0.85 [g]		0.60 [g]		0.03 [g]		0.06 [g]		<0.01 [g]
0	158	0		0		0		0		0		0	
1-5 cigarettes/day	20	0.10	0.72	0.04	0.93	-0.31	0.56	-0.94	0.05	-0.06	0.80	-0.33	0.56
6-10 cigarettes/day	8	-0.25	0.63	0.26	0.76	-0.52	0.32	0.30	0.54	-0.03	0.87	-0.67	0.45
≥ 10 cigarettes/day	5	0.58	0.12	-0.66	0.43	0.70	0.66	-1.5	0.08	0.53	0.02	3.5	<0.01
P trend			0.66		0.87		0.79		0.05		0.57		0.40
Continuous [f]		0.02	0.56	-0.02	0.68	-0.005	0.93	-0.10	0.02	0.02	0.33	0.08	0.24

[a] Regression parameters given for an increase by 1 year.
[b] Before pregnancy.
[c] Regression parameters are given for an increase by 1 kg/ m² in body mass index.
[d] Regression parameters are given for each additional child.
[e] Occupation at time collection of urine sample (yes/no).
[f] Regression parameters are given for an increase by one cigarette/day.
[g] *P-value* of heterogeneity test

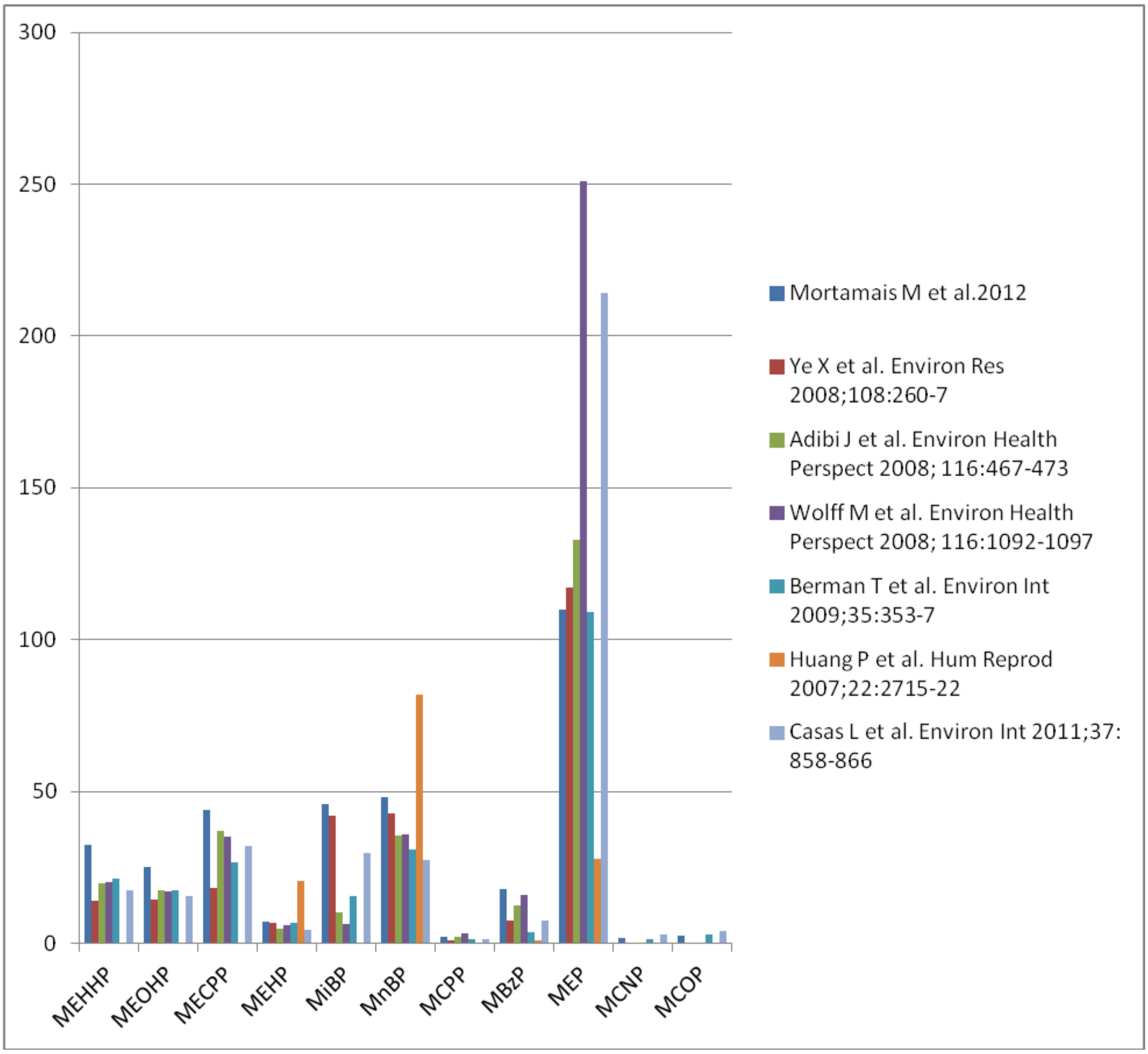

Figure 1, Supplemental Material. Median Values of Urinary Phthalate Metabolites (µg/l) in Selected Publications Among Pregnant Women.

Remark: The concentrations of monoethyl phthalate (MEP) and monobenzyl phthalate (MBzP) have been corrected for some studies; correction factors are 0.66 (MEP) and 0.72 (MBzP) (A. Calafat, personal communication). These correction factors have been applied to all of the studies that had their analyses done at CDC (Mortamais et al. 2012, Adibi et al. 2008, Wolff et al. 2008, Berman et al. 2009, and Casas et al. 2011).

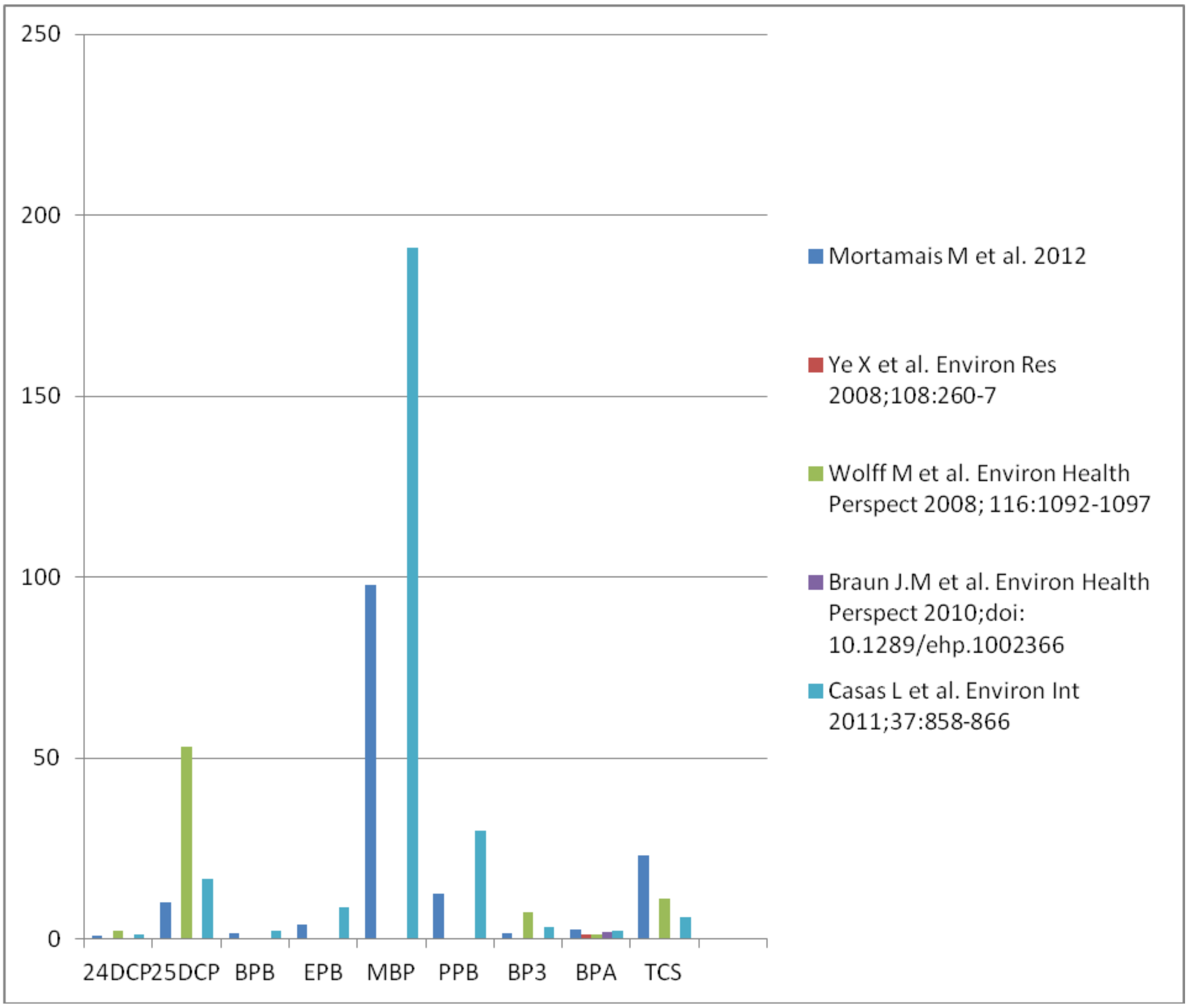

Figure 2, Supplemental Material. Median Values of Urinary Phenols (µg/l) in Selected Publications Among Pregnant Women.
